# 2-[3-(4-Chloro­phen­yl)-5-(4-fluoro­phenyl)-4,5-dihydro-1*H*-pyrazol-1-yl]-4-phenyl-1,3-thia­zole

**DOI:** 10.1107/S1600536813007496

**Published:** 2013-03-23

**Authors:** Bakr F. Abdel-Wahab, Seik Weng Ng, Edward R. T. Tiekink

**Affiliations:** a Applied Organic Chemistry Department, National Research Centre, Dokki, 12622 Giza, Egypt; b Department of Chemistry, University of Malaya, 50603 Kuala Lumpur, Malaysia; c Chemistry Department, Faculty of Science, King Abdulaziz University, PO Box 80203 Jeddah, Saudi Arabia

## Abstract

In the title compound, C_24_H_17_ClFN_3_S, the pyrazole ring is almost planar (r.m.s. deviation = 0.030 Å). With the exception of the methine-bound benzene ring, which forms a dihedral angle of 85.77 (13)° with the pyrazole ring, the remaining non-C atoms lie in an approximate plane (r.m.s. deviation = 0.084 Å) so that overall the mol­ecule has a T-shape. In the crystal, centrosymmetrically related mol­ecules are connected *via* π–π inter­actions between pyrazole rings [centroid–centroid distance = 3.5370 (15) Å] and these stack along the *a* axis with no specific inter­actions between them.

## Related literature

For the biological activity of pyrazolin-1-carbothio­amides, see: Abdel-Wahab *et al.* (2009 ▶, 2012 ▶); Lv *et al.* (2011 ▶); Chimenti *et al.* (2010 ▶). For a related structure, see: Abdel-Wahab *et al.* (2013 ▶).
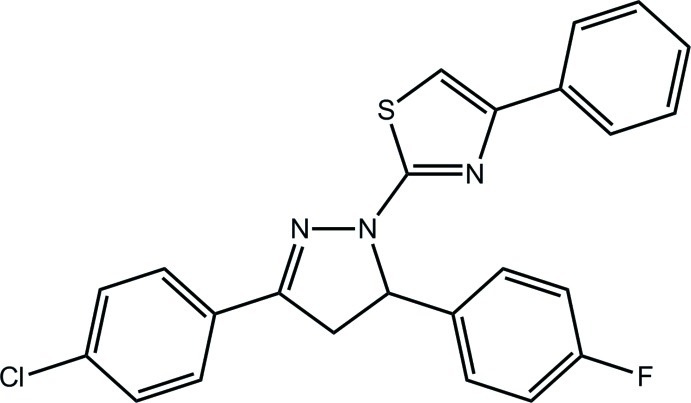


## Experimental

### 

#### Crystal data

C_24_H_17_ClFN_3_S
*M* *_r_* = 433.92
Monoclinic, 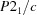

*a* = 11.1360 (9) Å
*b* = 16.4129 (16) Å
*c* = 11.6066 (7) Å
β = 98.170 (7)°
*V* = 2099.9 (3) Å^3^
*Z* = 4
Mo *K*α radiation
μ = 0.31 mm^−1^
*T* = 295 K
0.25 × 0.25 × 0.25 mm

#### Data collection

Agilent SuperNova Dual diffractometer with an Atlas detector
Absorption correction: multi-scan (*CrysAlis PRO*; Agilent, 2011 ▶) *T*_min_ = 0.956, *T*_max_ = 1.000
11642 measured reflections
4850 independent reflections
2627 reflections with *I* > 2σ(*I*)
*R*_int_ = 0.043

#### Refinement

*R*[*F* ^2^ > 2σ(*F* ^2^)] = 0.053
*wR*(*F* ^2^) = 0.140
*S* = 1.03
4850 reflections
271 parameters
H-atom parameters constrained
Δρ_max_ = 0.17 e Å^−3^
Δρ_min_ = −0.28 e Å^−3^

### 

Data collection: *CrysAlis PRO* (Agilent, 2011 ▶); cell refinement: *CrysAlis PRO*; data reduction: *CrysAlis PRO*; program(s) used to solve structure: *SHELXS97* (Sheldrick, 2008 ▶); program(s) used to refine structure: *SHELXL97* (Sheldrick, 2008 ▶); molecular graphics: *ORTEP-3 for Windows* (Farrugia, 2012 ▶) and *DIAMOND* (Brandenburg, 2006 ▶); software used to prepare material for publication: *publCIF* (Westrip, 2010 ▶).

## Supplementary Material

Crystal structure: contains datablock(s) global, I. DOI: 10.1107/S1600536813007496/hb7058sup1.cif

Structure factors: contains datablock(s) I. DOI: 10.1107/S1600536813007496/hb7058Isup2.hkl

Supplementary material file. DOI: 10.1107/S1600536813007496/hb7058Isup3.cml

Additional supplementary materials:  crystallographic information; 3D view; checkCIF report

## Figures and Tables

**Fig. 1.**
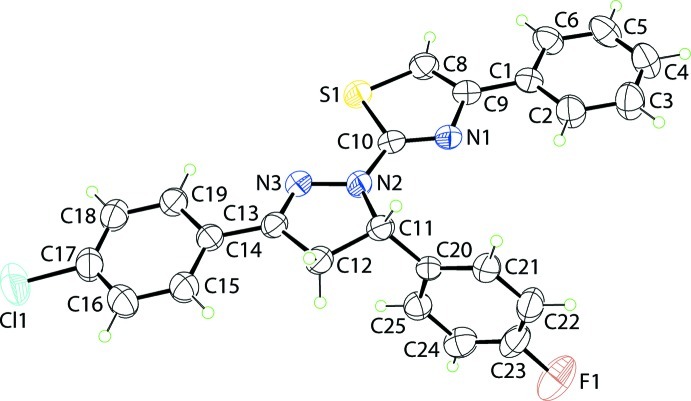
The molecular structure of (I) showing displacement ellipsoids at the 35% probability level.

**Fig. 2.**
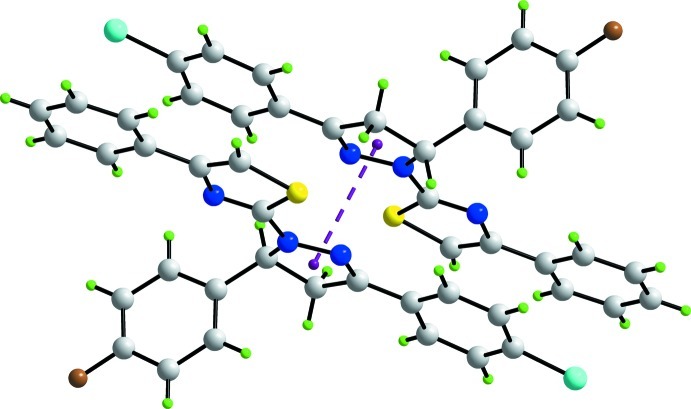
A view of the dimeric aggregate in (I) sustained by π—π interactions, shown as purple dashed lines.

**Fig. 3.**
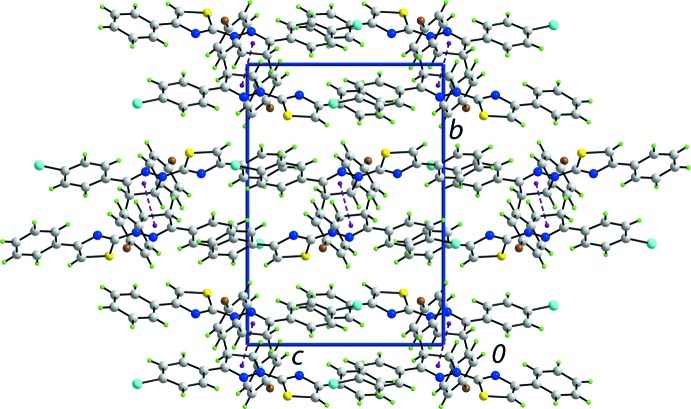
A view of the crystal packing in projection down the a axis. The π—π interactions are shown as purple dashed lines.

## supplementary crystallographic information

### Comment

The title compound (I) was investigated owing to the established biological activities exhibited by related pyrazolin-1-carbothioamides (Abdel-Wahab *et al.* 2012; Lv *et al.*, 2011; Chimenti *et al.*, 2010; Abdel-Wahab *et al.* 2009). Herein, the crystal and molecular structure of (I) is described.

In (I), Fig. 1, the pyrazolyl ring is quite planar with a r.m.s. deviation of the five atoms being 0.030 Å. This is in contrast to the situation observed in the recently described derivative with a methyl rather than a chloride (Abdel-Wahab *et al.* 2013) whereby an envelope conformation was found for each of the two independent molecules; the methine-C atom was the flap atom in each case. In (I), the dihedral angle between the pyrazolyl and thiazole (r.m.s. deviation = 0.002 Å) rings is 2.83 (13)°. The thiazole-bound benzene ring is co-planar; dihedral angle = 4.34 (13)° with the thiazole. About the pyrazolyl ring, the chlorobenzene ring is co-planar, dihedral angle = 6.92 (14)°, but the benzene ring bound at C11 is perpendicular, dihedral angle = 85.77 (13)°. Thus, there are two planar, mutually perpendicular domains in the molecule which adopts a T-shape, as was the case for the aforementioned literature structure but which exhibited some twists, *e.g*. between the five-membered rings (Abdel-Wahab *et al.* 2013).

The most prominent feature of the crystal packing is the formation of dimeric aggregates between centrosymmetrically related molecules *via*π—π interactions between pyrazolyl rings [inter-centroid distance = 3.5370 (15) Å for symmetry operation: 1 - *x*, 1 - *y*, 1 - *z*], Fig. 2. Dimeric units stack along the *a* axis with no specific interactions between them, Fig. 3.

### Experimental

A mixture of 3-(4-chlorophenyl)-5-(4-fluorophenyl)-4,5-dihydro-1*H*-pyrazole-1-carbothioamide (0.333 g, 0.001 *M*) and phenacyl bromide (0.2 g, 0.001 *M*) in anhydrous ethanol (30 ml) was heated under reflux for about 4 h. The resultant solid was filtered and dried. Recrystallization was by slow evaporation of an ethanol solution of (I) to yield yellow cubes in 55% yield; *M*.pt: 418–419 K.

### Refinement

Carbon-bound H-atoms were placed in calculated positions (C—H = 0.93 to 0.98 Å) and were included in the refinement in the riding model approximation, with *U*_iso_(H) = 1.2*U*_equiv_(C).

### Crystal data

**Table:**

C_24_H_17_ClFN_3_S	*F*(000) = 896
*M**_r_* = 433.92	*D*_x_ = 1.373 Mg m^−^^3^
Monoclinic, *P*2_1_/*c*	Mo *K*α radiation, λ = 0.71073 Å
Hall symbol: -P 2ybc	Cell parameters from 2134 reflections
*a* = 11.1360 (9) Å	θ = 3.0–27.5°
*b* = 16.4129 (16) Å	µ = 0.31 mm^−^^1^
*c* = 11.6066 (7) Å	*T* = 295 K
β = 98.170 (7)°	Cube, yellow
*V* = 2099.9 (3) Å^3^	0.25 × 0.25 × 0.25 mm
*Z* = 4	

### Data collection

**Table:**

Agilent SuperNova Dual diffractometer with an Atlas detector	4850 independent reflections
Radiation source: SuperNova (Mo) X-ray Source	2627 reflections with *I* > 2σ(*I*)
Mirror monochromator	*R*_int_ = 0.043
Detector resolution: 10.4041 pixels mm^-1^	θ_max_ = 27.6°, θ_min_ = 3.0°
ω scan	*h* = −14→13
Absorption correction: multi-scan (*CrysAlis PRO*; Agilent, 2011)	*k* = −21→21
*T*_min_ = 0.956, *T*_max_ = 1.000	*l* = −15→14
11642 measured reflections	

### Refinement

**Table:**

Refinement on *F*^2^	Primary atom site location: structure-invariant direct methods
Least-squares matrix: full	Secondary atom site location: difference Fourier map
*R*[*F*^2^ > 2σ(*F*^2^)] = 0.053	Hydrogen site location: inferred from neighbouring sites
*wR*(*F*^2^) = 0.140	H-atom parameters constrained
*S* = 1.03	*w* = 1/[σ^2^(*F*_o_^2^) + (0.0448*P*)^2^ + 0.1993*P*] where *P* = (*F*_o_^2^ + 2*F*_c_^2^)/3
4850 reflections	(Δ/σ)_max_ = 0.001
271 parameters	Δρ_max_ = 0.17 e Å^−^^3^
0 restraints	Δρ_min_ = −0.28 e Å^−^^3^

### Special details

**Table:**

Geometry. All e.s.d.'s (except the e.s.d. in the dihedral angle between two l.s. planes) are estimated using the full covariance matrix. The cell e.s.d.'s are taken into account individually in the estimation of e.s.d.'s in distances, angles and torsion angles; correlations between e.s.d.'s in cell parameters are only used when they are defined by crystal symmetry. An approximate (isotropic) treatment of cell e.s.d.'s is used for estimating e.s.d.'s involving l.s. planes.
Refinement. Refinement of *F*^2^ against ALL reflections. The weighted *R*-factor *wR* and goodness of fit *S* are based on *F*^2^, conventional *R*-factors *R* are based on *F*, with *F* set to zero for negative *F*^2^. The threshold expression of *F*^2^ > σ(*F*^2^) is used only for calculating *R*-factors(gt) *etc*. and is not relevant to the choice of reflections for refinement. *R*-factors based on *F*^2^ are statistically about twice as large as those based on *F*, and *R*- factors based on ALL data will be even larger.

### Fractional atomic coordinates and isotropic or equivalent isotropic displacement parameters (Å^2^)

**Table:**

	*x*	*y*	*z*	*U*_iso_*/*U*_eq_	
Cl1	0.78673 (9)	0.63985 (7)	1.06121 (7)	0.1183 (4)	
S1	0.51447 (6)	0.68324 (4)	0.29541 (5)	0.0663 (2)	
N1	0.30894 (17)	0.61006 (12)	0.23548 (15)	0.0540 (5)	
N2	0.38567 (17)	0.59931 (14)	0.43352 (16)	0.0635 (6)	
N3	0.48128 (17)	0.61670 (12)	0.51903 (16)	0.0576 (5)	
C1	0.2574 (2)	0.64048 (14)	0.02591 (19)	0.0550 (6)	
C2	0.1497 (3)	0.59731 (18)	0.0197 (2)	0.0774 (8)	
H2	0.1309	0.5703	0.0852	0.093*	
C3	0.0700 (3)	0.5940 (2)	−0.0830 (2)	0.0925 (10)	
H3	−0.0015	0.5643	−0.0863	0.111*	
C4	0.0959 (3)	0.63419 (19)	−0.1797 (2)	0.0851 (9)	
H4	0.0421	0.6318	−0.2487	0.102*	
C5	0.2002 (3)	0.67765 (18)	−0.1749 (2)	0.0817 (9)	
H5	0.2173	0.7057	−0.2403	0.098*	
C6	0.2807 (3)	0.68030 (16)	−0.0734 (2)	0.0708 (8)	
H6	0.3525	0.7096	−0.0716	0.085*	
C8	0.4471 (2)	0.68840 (16)	0.1533 (2)	0.0649 (7)	
H8	0.4796	0.7162	0.0951	0.078*	
C9	0.3406 (2)	0.64703 (14)	0.13609 (19)	0.0544 (6)	
C10	0.3922 (2)	0.62494 (14)	0.32298 (19)	0.0528 (6)	
C11	0.2942 (2)	0.54327 (15)	0.47086 (19)	0.0555 (6)	
H11	0.2985	0.4907	0.4317	0.067*	
C12	0.3456 (2)	0.53387 (16)	0.60180 (19)	0.0600 (7)	
H12A	0.2901	0.5562	0.6509	0.072*	
H12B	0.3609	0.4771	0.6220	0.072*	
C13	0.4618 (2)	0.58159 (15)	0.6141 (2)	0.0537 (6)	
C14	0.5449 (2)	0.59300 (15)	0.72236 (19)	0.0544 (6)	
C15	0.5162 (2)	0.56281 (17)	0.8256 (2)	0.0708 (7)	
H15	0.4455	0.5328	0.8258	0.085*	
C16	0.5917 (3)	0.5766 (2)	0.9296 (2)	0.0829 (9)	
H16	0.5713	0.5563	0.9991	0.099*	
C17	0.6964 (3)	0.62029 (19)	0.9294 (2)	0.0748 (8)	
C18	0.7284 (2)	0.64998 (17)	0.8275 (2)	0.0719 (8)	
H18	0.8003	0.6788	0.8279	0.086*	
C19	0.6529 (2)	0.63669 (16)	0.7247 (2)	0.0624 (7)	
H19	0.6742	0.6571	0.6556	0.075*	
C20	0.1672 (2)	0.57571 (15)	0.44605 (17)	0.0513 (6)	
C21	0.0808 (2)	0.53493 (17)	0.3704 (2)	0.0658 (7)	
H21	0.1030	0.4882	0.3333	0.079*	
C22	−0.0367 (3)	0.5615 (2)	0.3486 (2)	0.0814 (9)	
H22	−0.0942	0.5334	0.2975	0.098*	
C23	−0.0668 (2)	0.6295 (2)	0.4033 (3)	0.0812 (9)	
C24	0.0139 (3)	0.67373 (18)	0.4776 (2)	0.0763 (8)	
H24	−0.0096	0.7208	0.5130	0.092*	
C25	0.1323 (2)	0.64571 (16)	0.4982 (2)	0.0626 (7)	
H25	0.1895	0.6747	0.5482	0.075*	
F1	−0.18491 (17)	0.65571 (14)	0.3846 (2)	0.1352 (8)	

### Atomic displacement parameters (Å^2^)

**Table:**

	*U*^11^	*U*^22^	*U*^33^	*U*^12^	*U*^13^	*U*^23^
Cl1	0.1101 (7)	0.1603 (10)	0.0746 (5)	−0.0153 (6)	−0.0217 (5)	−0.0053 (5)
S1	0.0548 (4)	0.0757 (5)	0.0696 (4)	−0.0128 (3)	0.0131 (3)	0.0027 (3)
N1	0.0499 (12)	0.0621 (13)	0.0519 (11)	−0.0029 (9)	0.0140 (9)	0.0029 (9)
N2	0.0474 (12)	0.0907 (16)	0.0525 (11)	−0.0160 (11)	0.0075 (9)	0.0026 (10)
N3	0.0467 (12)	0.0678 (14)	0.0590 (11)	−0.0024 (10)	0.0097 (9)	−0.0021 (10)
C1	0.0615 (16)	0.0520 (15)	0.0537 (13)	−0.0006 (12)	0.0155 (11)	0.0003 (11)
C2	0.082 (2)	0.086 (2)	0.0635 (16)	−0.0220 (17)	0.0072 (14)	0.0121 (14)
C3	0.090 (2)	0.108 (3)	0.0753 (19)	−0.0319 (19)	−0.0035 (17)	0.0120 (17)
C4	0.103 (3)	0.089 (2)	0.0588 (16)	−0.0135 (19)	−0.0031 (16)	−0.0019 (15)
C5	0.114 (3)	0.081 (2)	0.0521 (15)	−0.0105 (19)	0.0169 (16)	0.0027 (14)
C6	0.085 (2)	0.0721 (19)	0.0583 (15)	−0.0137 (15)	0.0200 (14)	0.0003 (13)
C8	0.0638 (17)	0.0667 (18)	0.0669 (15)	−0.0105 (13)	0.0190 (13)	0.0060 (13)
C9	0.0568 (15)	0.0511 (15)	0.0579 (13)	0.0013 (12)	0.0172 (12)	−0.0001 (11)
C10	0.0464 (14)	0.0572 (15)	0.0575 (13)	0.0015 (11)	0.0170 (11)	0.0014 (11)
C11	0.0488 (14)	0.0612 (16)	0.0580 (13)	−0.0038 (11)	0.0133 (11)	−0.0046 (11)
C12	0.0509 (15)	0.0692 (18)	0.0602 (14)	0.0021 (12)	0.0083 (11)	0.0079 (12)
C13	0.0457 (14)	0.0577 (15)	0.0582 (13)	0.0046 (11)	0.0090 (11)	0.0011 (12)
C14	0.0530 (15)	0.0545 (15)	0.0559 (13)	0.0088 (12)	0.0080 (11)	−0.0006 (11)
C15	0.0639 (17)	0.084 (2)	0.0639 (15)	−0.0053 (14)	0.0065 (13)	0.0087 (14)
C16	0.086 (2)	0.102 (3)	0.0598 (16)	−0.0041 (18)	0.0054 (15)	0.0133 (15)
C17	0.0692 (19)	0.083 (2)	0.0674 (17)	0.0049 (16)	−0.0061 (14)	−0.0028 (15)
C18	0.0583 (17)	0.076 (2)	0.0782 (18)	−0.0008 (14)	0.0007 (14)	−0.0073 (15)
C19	0.0575 (16)	0.0656 (17)	0.0639 (14)	0.0014 (13)	0.0078 (12)	−0.0024 (12)
C20	0.0474 (14)	0.0630 (16)	0.0448 (11)	−0.0062 (11)	0.0110 (10)	0.0028 (11)
C21	0.0568 (16)	0.0739 (19)	0.0666 (15)	−0.0072 (13)	0.0078 (12)	−0.0090 (13)
C22	0.0596 (19)	0.097 (3)	0.0828 (19)	−0.0129 (16)	−0.0055 (15)	0.0028 (17)
C23	0.0462 (17)	0.096 (2)	0.100 (2)	0.0062 (16)	0.0039 (16)	0.0247 (19)
C24	0.073 (2)	0.0684 (19)	0.0896 (19)	0.0136 (15)	0.0176 (16)	0.0093 (15)
C25	0.0616 (17)	0.0636 (18)	0.0627 (14)	−0.0039 (13)	0.0087 (13)	0.0018 (13)
F1	0.0629 (12)	0.1386 (19)	0.197 (2)	0.0270 (11)	−0.0043 (13)	0.0207 (15)

### Geometric parameters (Å, º)

**Table:**

Cl1—C17	1.737 (2)	C12—C13	1.501 (3)
S1—C8	1.714 (2)	C12—H12A	0.9700
S1—C10	1.731 (2)	C12—H12B	0.9700
N1—C10	1.297 (3)	C13—C14	1.463 (3)
N1—C9	1.392 (3)	C14—C15	1.376 (3)
N2—C10	1.362 (3)	C14—C19	1.398 (3)
N2—N3	1.378 (2)	C15—C16	1.388 (3)
N2—C11	1.482 (3)	C15—H15	0.9300
N3—C13	1.290 (3)	C16—C17	1.369 (4)
C1—C6	1.381 (3)	C16—H16	0.9300
C1—C2	1.386 (3)	C17—C18	1.373 (4)
C1—C9	1.472 (3)	C18—C19	1.375 (3)
C2—C3	1.382 (3)	C18—H18	0.9300
C2—H2	0.9300	C19—H19	0.9300
C3—C4	1.368 (4)	C20—C21	1.380 (3)
C3—H3	0.9300	C20—C25	1.380 (3)
C4—C5	1.358 (4)	C21—C22	1.368 (4)
C4—H4	0.9300	C21—H21	0.9300
C5—C6	1.376 (3)	C22—C23	1.350 (4)
C5—H5	0.9300	C22—H22	0.9300
C6—H6	0.9300	C23—C24	1.363 (4)
C8—C9	1.357 (3)	C23—F1	1.371 (3)
C8—H8	0.9300	C24—C25	1.384 (4)
C11—C20	1.500 (3)	C24—H24	0.9300
C11—C12	1.554 (3)	C25—H25	0.9300
C11—H11	0.9800		
			
C8—S1—C10	87.52 (12)	C13—C12—H12B	111.1
C10—N1—C9	109.26 (19)	C11—C12—H12B	111.1
C10—N2—N3	118.38 (19)	H12A—C12—H12B	109.0
C10—N2—C11	126.72 (18)	N3—C13—C14	121.0 (2)
N3—N2—C11	114.24 (17)	N3—C13—C12	113.54 (19)
C13—N3—N2	108.45 (19)	C14—C13—C12	125.4 (2)
C6—C1—C2	117.5 (2)	C15—C14—C19	118.2 (2)
C6—C1—C9	121.4 (2)	C15—C14—C13	120.4 (2)
C2—C1—C9	121.0 (2)	C19—C14—C13	121.3 (2)
C3—C2—C1	120.7 (3)	C14—C15—C16	120.8 (3)
C3—C2—H2	119.7	C14—C15—H15	119.6
C1—C2—H2	119.7	C16—C15—H15	119.6
C4—C3—C2	120.3 (3)	C17—C16—C15	119.6 (3)
C4—C3—H3	119.9	C17—C16—H16	120.2
C2—C3—H3	119.9	C15—C16—H16	120.2
C5—C4—C3	119.9 (3)	C16—C17—C18	120.8 (2)
C5—C4—H4	120.0	C16—C17—Cl1	118.8 (2)
C3—C4—H4	120.0	C18—C17—Cl1	120.3 (2)
C4—C5—C6	120.1 (3)	C17—C18—C19	119.3 (3)
C4—C5—H5	120.0	C17—C18—H18	120.3
C6—C5—H5	120.0	C19—C18—H18	120.3
C5—C6—C1	121.5 (3)	C18—C19—C14	121.1 (3)
C5—C6—H6	119.2	C18—C19—H19	119.5
C1—C6—H6	119.2	C14—C19—H19	119.5
C9—C8—S1	111.78 (19)	C21—C20—C25	117.9 (2)
C9—C8—H8	124.1	C21—C20—C11	120.2 (2)
S1—C8—H8	124.1	C25—C20—C11	121.9 (2)
C8—C9—N1	114.5 (2)	C22—C21—C20	121.7 (3)
C8—C9—C1	126.4 (2)	C22—C21—H21	119.2
N1—C9—C1	119.1 (2)	C20—C21—H21	119.2
N1—C10—N2	123.5 (2)	C23—C22—C21	118.1 (3)
N1—C10—S1	116.92 (17)	C23—C22—H22	121.0
N2—C10—S1	119.57 (17)	C21—C22—H22	121.0
N2—C11—C20	113.08 (19)	C22—C23—C24	123.5 (3)
N2—C11—C12	100.03 (17)	C22—C23—F1	118.8 (3)
C20—C11—C12	115.44 (19)	C24—C23—F1	117.6 (3)
N2—C11—H11	109.3	C23—C24—C25	117.3 (3)
C20—C11—H11	109.3	C23—C24—H24	121.4
C12—C11—H11	109.3	C25—C24—H24	121.4
C13—C12—C11	103.54 (19)	C20—C25—C24	121.4 (2)
C13—C12—H12A	111.1	C20—C25—H25	119.3
C11—C12—H12A	111.1	C24—C25—H25	119.3
			
C10—N2—N3—C13	−174.7 (2)	N2—N3—C13—C14	−176.4 (2)
C11—N2—N3—C13	−3.4 (3)	N2—N3—C13—C12	0.5 (3)
C6—C1—C2—C3	0.7 (4)	C11—C12—C13—N3	2.3 (3)
C9—C1—C2—C3	177.6 (3)	C11—C12—C13—C14	179.0 (2)
C1—C2—C3—C4	−0.8 (5)	N3—C13—C14—C15	172.7 (2)
C2—C3—C4—C5	−0.1 (5)	C12—C13—C14—C15	−3.8 (4)
C3—C4—C5—C6	0.9 (5)	N3—C13—C14—C19	−5.3 (4)
C4—C5—C6—C1	−1.0 (4)	C12—C13—C14—C19	178.2 (2)
C2—C1—C6—C5	0.2 (4)	C19—C14—C15—C16	1.1 (4)
C9—C1—C6—C5	−176.7 (2)	C13—C14—C15—C16	−176.9 (2)
C10—S1—C8—C9	−0.2 (2)	C14—C15—C16—C17	−0.5 (5)
S1—C8—C9—N1	0.2 (3)	C15—C16—C17—C18	−0.6 (5)
S1—C8—C9—C1	178.26 (19)	C15—C16—C17—Cl1	177.8 (2)
C10—N1—C9—C8	0.0 (3)	C16—C17—C18—C19	1.1 (4)
C10—N1—C9—C1	−178.2 (2)	Cl1—C17—C18—C19	−177.3 (2)
C6—C1—C9—C8	−2.2 (4)	C17—C18—C19—C14	−0.5 (4)
C2—C1—C9—C8	−178.9 (3)	C15—C14—C19—C18	−0.6 (4)
C6—C1—C9—N1	175.8 (2)	C13—C14—C19—C18	177.4 (2)
C2—C1—C9—N1	−0.9 (4)	N2—C11—C20—C21	117.2 (2)
C9—N1—C10—N2	177.8 (2)	C12—C11—C20—C21	−128.4 (2)
C9—N1—C10—S1	−0.2 (3)	N2—C11—C20—C25	−63.6 (3)
N3—N2—C10—N1	178.1 (2)	C12—C11—C20—C25	50.8 (3)
C11—N2—C10—N1	8.0 (4)	C25—C20—C21—C22	−1.2 (4)
N3—N2—C10—S1	−3.9 (3)	C11—C20—C21—C22	178.0 (2)
C11—N2—C10—S1	−174.08 (19)	C20—C21—C22—C23	0.1 (4)
C8—S1—C10—N1	0.3 (2)	C21—C22—C23—C24	1.0 (5)
C8—S1—C10—N2	−177.8 (2)	C21—C22—C23—F1	−178.4 (3)
C10—N2—C11—C20	−61.7 (3)	C22—C23—C24—C25	−1.0 (5)
N3—N2—C11—C20	127.8 (2)	F1—C23—C24—C25	178.4 (2)
C10—N2—C11—C12	175.0 (2)	C21—C20—C25—C24	1.3 (4)
N3—N2—C11—C12	4.5 (3)	C11—C20—C25—C24	−177.9 (2)
N2—C11—C12—C13	−3.7 (2)	C23—C24—C25—C20	−0.2 (4)
C20—C11—C12—C13	−125.4 (2)		
